# Extended reality in cranial and spinal neurosurgery – a bibliometric analysis

**DOI:** 10.1007/s00701-024-06072-4

**Published:** 2024-04-25

**Authors:** Ali Buwaider, Victor Gabriel El-Hajj, Omar Ali Mahdi, Alessandro Iop, Maria Gharios, Andrea de Giorgio, Mario Romero, Paul Gerdhem, Walter C Jean, Erik Edström, Adrian Elmi-Terander

**Affiliations:** 1 Department of Clinical Neuroscience, Karolinska Institutet, Stockholm, Sweden; 2 KTH Royal Institute of Technology, Stockholm, Sweden; 3 Artificial Engineering, Naples, Italy; 4 Department of Orthopaedics and Hand surgery, Uppsala University hospital, Uppsala, Sweden; 5 Department of Surgical Sciences, Uppsala University, Uppsala, Sweden; 6 Division of Neurosurgery, Lehigh Valley Fleming Neuroscience Institute, Allentown, PA USA; 7 Department of Neurosurgery & Brain Repair, Morsani College of Medicine, University of South Florida, Tampa, FL USA; 8 Capio Spine Center Stockholm, Löwenströmska Hospital, Upplands-Väsby, Sweden; 9 Department of Medical Sciences, Örebro University, Örebro, Sweden

**Keywords:** Extended reality, Virtual reality, Augmented reality, Mixed reality, Neurosurgery, Bibliometrics

## Abstract

**Purpose:**

This bibliometric analysis of the top 100 cited articles on extended reality (XR) in neurosurgery aimed to reveal trends in this research field. Gender differences in authorship and global distribution of the most-cited articles were also addressed.

**Methods:**

A Web of Science electronic database search was conducted. The top 100 most-cited articles related to the scope of this review were retrieved and analyzed for trends in publications, journal characteristics, authorship, global distribution, study design, and focus areas. After a brief description of the top 100 publications, a comparative analysis between spinal and cranial publications was performed.

**Results:**

From 2005, there was a significant increase in spinal neurosurgery publications with a focus on pedicle screw placement. Most articles were original research studies, with an emphasis on augmented reality (AR). In cranial neurosurgery, there was no notable increase in publications. There was an increase in studies assessing both AR and virtual reality (VR) research, with a notable emphasis on VR compared to AR. Education, surgical skills assessment, and surgical planning were more common themes in cranial studies compared to spinal studies. Female authorship was notably low in both groups, with no significant increase over time. The USA and Canada contributed most of the publications in the research field.

**Conclusions:**

Research regarding the use of XR in neurosurgery increased significantly from 2005. Cranial research focused on VR and resident education while spinal research focused on AR and neuronavigation. Female authorship was underrepresented. North America provides most of the high-impact research in this area.

**Supplementary information:**

The online version contains supplementary material available at 10.1007/s00701-024-06072-4.

## Introduction

Within neurosurgery, extended reality (XR) has experienced a substantial growth over the past decade [7]. XR serves as a comprehensive term referring to virtual reality (VR), augmented reality (AR), and mixed reality (MR) [24, 41]. These systems provide a varying degree of immersive three-dimensional (3D) virtual imaging experiences [41]. VR provides a fully immersive experience where the user enters a completely virtual environment, often without interacting with real world objects [41]. AR and MR blend virtual elements into the real world [41]. While AR solely overlays virtual elements onto real objects, MR enables dynamic interactions between the overlayed virtual elements and the real environment [41].

Several neurosurgical centers have reported various benefits of using XR [7, 8, 17, 31, 32]. For instance, XR complements existing neuronavigation systems in situations when two-dimensional navigation is limiting [21, 31], and the precision of XR-based navigation systems have been demonstrated in the treatment of intracranial pathologies such as aneurysms, gliomas, and meningiomas [29]. Another application is in the training of neurosurgical residents [5, 8]. XR-based training offers an interactive surgical experience, integrating visualization of anatomy and haptic feedback [24]. This training modality reinforces residents' procedural memory and confidence, reducing the time needed to perform a surgery [12]. Other reported benefits of XR-based training include reduced radiation exposure, lower rates of revision surgeries, and an overall improvement in the safety and precision of surgical procedures [7, 8, 17, 31, 32].

Despite existing bibliometric analyses on the use of XR in surgical procedures [22, 42], none have specifically targeted the field of neurosurgery. Some systematic reviews have tried to integrate some type of literature analysis on the use of XR in neurosurgery [11, 24, 25], but none have provided a comprehensive analysis of current literature. This bibliometric analysis aimed to detect trends among the top 100 cited articles on XR utilization in neurosurgery. Given the international disparities in determining which surgeons should manage spinal cases [3], trends were separately presented for cranial and spinal procedures. Differences between groups were delineated and discussed. In line with the current emphasis on addressing gender disparities in authorships of neurosurgical publications [4], the analysis described the relative contributions of female authors to the current literature. Additionally, global distribution and international collaborations were addressed.

## Methods and material

### Search strategy and study selection

As of July 2023, an electronic database search was performed on Web of Science using the following search strategy: ["augmented reality" OR "virtual reality" OR "extended reality" OR "mixed reality" OR "augmented virtuality"] AND ["spine" OR "cerebrovascular" OR "spinal" OR "lumbar" OR "cranial" OR "skull base" OR "pedicle screw" OR "pedicle screws" OR "neurovascular" OR "neuro-oncology" OR "brain tumor" OR "glioma"] AND ["neurosurgery" OR "surgery" OR "neurointervention" OR "neurointerventional" OR "neurosurgical" OR "brain surgery" OR "neurological surgery" OR "mapping"]. There were no limitations with respect to the language or year of publication. Results were subsequently sorted by citation count, and the top-100 most-cited articles relevant to the scope of this review were retrieved. The search was performed by two independently and discrepancies were later resolved by consensus.

### Data extraction

The following characteristics were analyzed: trends in publications, journal of publications, authorship, global distribution, study design and area of focus. 1) Trends of publications encompassed the number of publications per year, number of citations, number of citations per year, accessibility, and the top 10 most-cited articles. 2) Journal of publications encompassed journal name, impact factor, and the journals with the most publications. Impact factors were extracted from Thomson Reuters Journal Citation Reports. 3) Authorship included the names of the first and last authors and female authorship. 4) Global distribution encompassed the publications’ country of origin, affiliation, and international collaborations. For articles with authors from different countries or institutions, the first author's country and institution were noted. 5) Study design was presented regarding whether the study was a review or original research study. 6) The area of focus encompassed the type of XR used, whether head-mounted displays (HMDs) were used, study subjects, neurosurgical subspecialty (skull base, cerebrovascular, pediatric, neurosurgical oncology, spine, and endovascular), and the main use of XR. The top 100 publications were divided among spinal or cranial procedures. Only a brief description of the top 100 publications was provided while a detailed description was provided for spinal and cranial subgroups.

### Statistical analysis

The distribution of continuous data was assessed using the Shapiro-Wilk's test. For normally distributed data, mean values were presented with standard deviations, while non-normally distributed data was expressed as median with interquartile range (IQR). Statistical comparisons between spinal and cranial studies involved the use of Student’s t-test and Mann-Whitney U-test for continuous variables, and the χ2 test for categorical variables. Choropleth maps and hierarchical edge bundling were used for visual illustration of international collaborations. The Mann-Kendall’s test was employed across all variables to evaluate their trends over the years. Categorical variables were analyzed as proportions and continuous variables as averages. A *p*-value < 0.05 was considered statistically significant. All statistical analyses were conducted using R version 4.2.2 (R Foundation for Statistical Computing, Vienna, Austria) [36].

## Results

In accordance with the search strategy, 675 studies examining the application of XR in neurosurgery were identified. Among the top 100 cited articles, 47 studies focused on cranial procedures, 37 on spinal procedures, and 16 discussed both aspects (Supplemental Table 1).

**Fig. 1:**
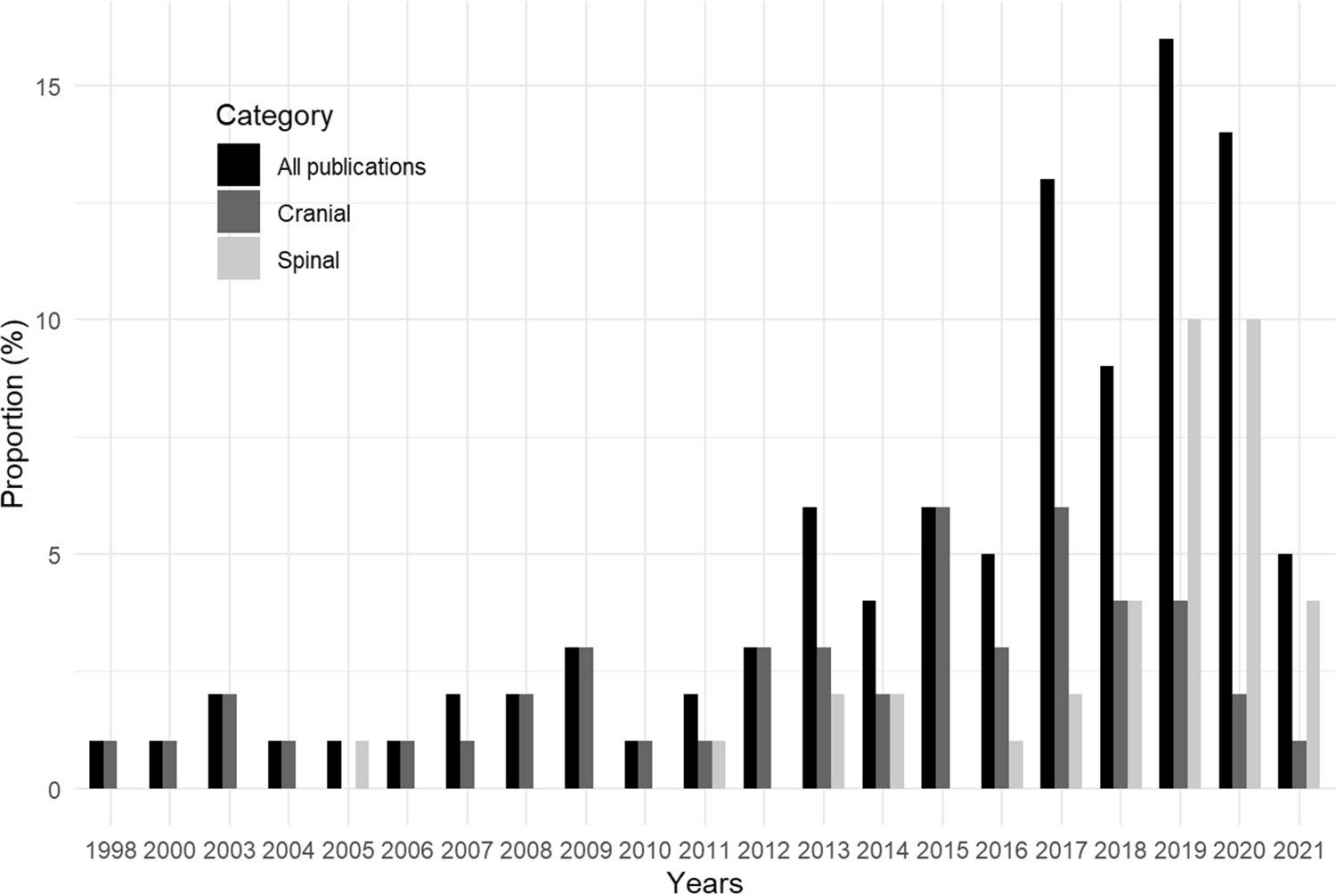
Proportional distribution of the top 100 most-cited publications on the use of extended reality in neurosurgery per year. The proportions of all publications are illustrated in black. Cranial only publications are illustrated in dark grey. Spinal only publications are illustrated in light grey

Since 1998, there has been a significant increase in the annual number of publications focusing on XR use in neurosurgery (*p*<0.001; Fig. 1). The 100 most-cited publications were published between 1998 and 2021. Yearly publications were not evenly distributed and 68% of articles were published after 2015. 40% were accessible as open access, with a discernible upward trajectory in open access articles (*p*<0.001). Most articles were original research (85%). Citation counts ranged from 22 to 191, with a median average yearly citation count of 5.8 (IQR 3.52 – 8.81). There was no statistically significant increase in average yearly citations (*p*=0.164). The top 100 publications involved contributions from 61 different journals, 61 institutions, 85 first authors, and 80 last authors (Table 1).

**Table 1:** Characteristics of the 100 top-cited studies

Variable	All studies (*N*=100)
Number of citations	
Median [Q1, Q3]	41.0 [30.0, 59.3]
Citations per year	
Median [Q1, Q3]	5.81 [3.52, 8.81]
Open access	33 (39%)
Journal Impact Factor	
Median [Q1, Q3]	3.40 [2.52, 5.30]
Female authorship	
First author	7 (7%)
Last author	1 (1%)
First author profession	
Engineer	16 (16%)
Neurosurgeon	59 (59%)
Orthopedic surgeon	5 (5.0%)
Last author profession	
Neurosurgeon	63 (63%)
Orthopedic surgeon	13 (13%)
Engineer	12 (12%)
Number of authors	
Mean (SD)	7.05 (3.0)
Article type	
Article	85 (85%)
Review	15 (15%)
Models used	
Physical 3D models	24 (27%)
Human subjects	25 (26%)
Virtual 3D models	22 (25%)
Not mentioned	26 (19%)
Cadavers	10 (11%)
Screen	2 (2.4%)
Type of XR	
AR	56 (57%)
VR	35 (36%)
MR	2 (2.4%)
Main use of XR	
Navigation	62 (62%)
Education and simulation	22 (22%)
Assessing surgical skills	14 (17%)
Surgical planning	14 (14%)
Patient education	1 (1%)
HMD	18 (18%)

Publications analyzed the use of AR (57%), VR (35%), and MR (2.4%). No significant increase in yearly publications regarding AR (*p*=0.087) or VR (*p*=0.120) solutions was observed. The use of HMDs for presenting virtual elements was detailed in 18 studies, with Microsoft HoloLens being the most frequently employed device (39%). The applications of XR spanned across neuronavigation (62%), surgical education (22%), assessment of surgical skills (17%), preoperative planning of surgical procedures (14%), and patient education (1%). The use of XR for assessing surgical skills (*p*=0.011) and surgical education (*p*=0.031) demonstrated an increasing trend over the years.

### Spinal only publications

Among the top 100 list, 37 studies exclusively addressed the spine. Since 2005, there has been a statistically significant increase in the number of publications focusing on the use of XR in spinal neurosurgery (*p*<0.001; Fig. 1). Nearly half were open access (49%). Citation counts of these studies ranged from 23 to 109, with a median count of 38.0 (IQR 29.0 – 58.0). The median number of average yearly citations was 5.8 (IQR 2.77 – 8.86). The most cited article, "A novel 3D guidance system using augmented reality for percutaneous vertebroplasty" by Abe et al. [1] was published in the Journal of Neurosurgery-Spine in 2013. The article with the highest number of citations per year was "Pedicle Screw Placement Using Augmented Reality Surgical Navigation with Intraoperative 3D Imaging: A First In-Human Prospective Cohort Study" by Elmi-Terander et al. [19] published in Spine in 2019 (Table 2). On average the article was cited 21 times yearly. A significant increase was detected in open access articles (*p*=0.002) and the average numbers of citations/year (*p*=0.040).

**Table 2:** Top 10 studies addressing the use of extended reality in spinal neurosurgery

Rank	Year	Title	Citation count	Citations per year	First Author	Country
1	2013	A novel 3D guidance system using augmented reality for percutaneous vertebroplasty	109	7.45	Abe	Japan
2	2019	Pedicle Screw Placement Using Augmented Reality Surgical Navigation with Intraoperative 3D Imaging: A First In-Human Prospective Cohort Study	106	21	Elmi-Terander	Sweden
3	2016	Surgical Navigation Technology Based on Augmented Reality and Integrated 3D Intraoperative Imaging: A Spine Cadaveric Feasibility and Accuracy Study	103	10	Elmi-Terander	Sweden
4	2019	Head-mounted display augmented reality to guide pedicle screw placement utilizing computed tomography	95	18.8	Gibby	USA
5	2018	Feasibility and Accuracy of Thoracolumbar Minimally Invasive Pedicle Screw Placement with Augmented Reality Navigation Technology	71	11.17	Elmi-Terander	Sweden
6	2011	Learning Retention of Thoracic Pedicle Screw Placement Using a High-Resolution Augmented Reality Simulator with Haptic Feedback	70	5.31	Luciano	USA
7	2017	Augmented reality surgical navigation with ultrasound-assisted registration for pedicle screw placement: a pilot study	67	8.86	Ma	China
8	2019	Augmented reality-assisted pedicle screw insertion: a cadaveric proof-of-concept study	59	11.4	Molina	USA
9	2014	Real-time advanced spinal surgery via visible patient model and augmented reality system	58	2.43	Wu	Taiwan
10	2014	Virtual reality spine surgery simulation: an empirical study of its usefulness	58	5.4	Gasco	USA

#### Journals

The top five journals with the highest number of published articles were Spine (*n*=5), World Neurosurgery (*n*=4), Journal of Neurosurgery – Spine (*n*=4), Operative Neurosurgery (*n*=3), and The Spine Journal (*n*=3), as outlined in Table 3.

**Table 3:** Top five authors, journals, and institutions

Spinal only	Number of publications	Cranial only	Number of publications
First authors			
Elmi-Terander, Adrian	4 (11%)	Kockro, Ralf A.	3 (6.4%)
Molina, Camilo A.	3 (8.1%)	Alotaibi, Fahad E.	2 (4.3%)
Burström, Gustav	3 (8.1%)	Azarnoush, Hamed	2 (4.3%)
Edström, Erik	2 (5.4%)	Winkler-Schwartz, Alexander	2 (4.3%)
Luciano, Cristian J.	2 (5.4%)	Stadie, Axel Thomas	2 (4.3%)
Last authors			
Elmi-Terander, Adrian	5 (14%)	Del Maestro, Rolando F.	11 (23%)
Roitberg, Ben Z.	3 (8.1%)	Samii, Madjid	2 (4.3%)
Del Maestro, Rolando F.	2 (5.4%)	-	-
Nimsky, Christopher	2 (5.4%)	-	-
Gerdhem, Paul	2 (5.4%)	-	-
Journals			
Spine	5 (14%)	Neurosurgery	9 (19%)
World Neurosurgery	4 (11%)	World Neurosurgery	6 (13%)
Journal of Neurosurgery – Spine	4 (11%)	International Journal of Computer Assisted Radiology and Surgery	4 (8.5%)
Operative Neurosurgery	3 (8.1%)	Journal Of Neurosurgery	3 (6.4%)
The Spine Journal	3 (8.1%)	Operative Neurosurgery	2 (4.3%)
Institutions			
Karolinska Institute	9 (24%)	McGill University	11 (23%)
Johns Hopkins University	2 (5.4%)	Johannes Gutenberg University of Mainz	3 (6.4%)
McGill University	2 (5.4%)	University of Pisa	2 (4.3%)
University of Illinois System	2 (5.4%)	University of Illinois System	2 (4.3%)
University of Zurich	2 (5.4%)	Fudan University	2 (4.3%)

#### Authorship

The topmost cited publications in spinal neurosurgery involved 28 first authors and 31 last authors. The top five first authors were Elmi-Terander (*n*=4), Molina (*n*=3), Burström (*n*=3), Edström (*n*=2), and Luciano (*n*=2). The top five last authors were Elmi-Terander (*n*=5), Roitberg (*n*=3), Gerdhem (*n*=2), Del Maestro (*n*=2), and Nimsky (*n*=2) (Table 3). The majority of first (51%) and last (49%) authors were neurosurgeons (Table 3). Other professions included orthopedic surgeons and engineers. The total number of authors per publication ranged from 2 to 16, with an average of 7.08 authors per article (SD 2.92). Female authorship was relatively limited with three female authors (two neurosurgeons and one orthopedic surgeon; 8.1%), listed as first and no females listed as last authors. There were no significant trends in the rate of female first authorship contribution over the years (*p*=0.137).

#### Global distribution

**Fig. 2:**
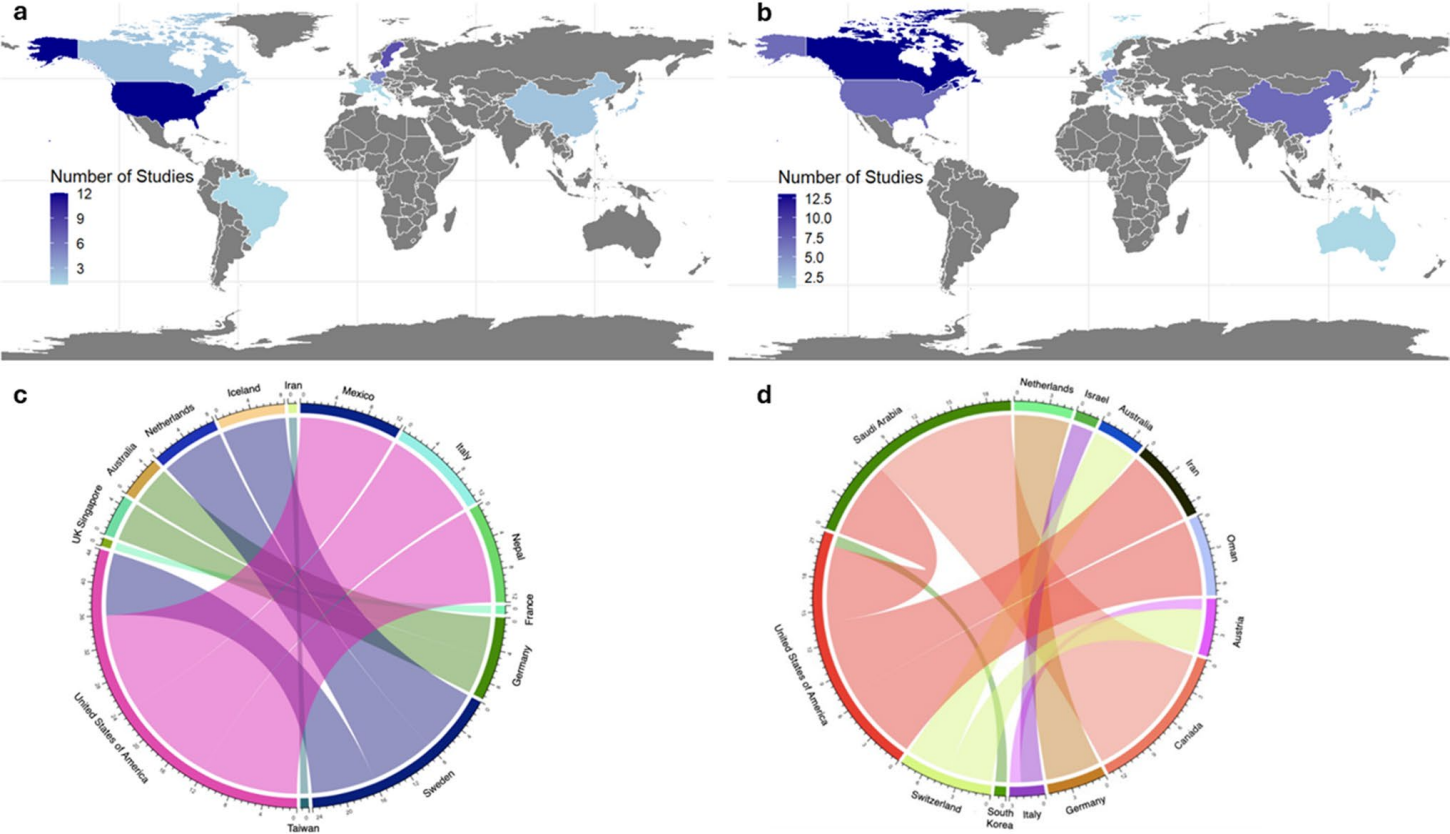
Choropleth map illustrating countries contributing to the application of XR in spinal (**a**) and cranial neurosurgery (**b**), as well as hierarchical edge bundle illustrating international collaborations on spinal (**c**) and cranial studies (**d**)

Research on the application of XR in spinal surgery involved twenty-four countries, with seven countries contributing two or more manuscripts among the list of the top cited spine publications (Fig. 2a). The USA was the leading country of origin (*n*=12), followed by Sweden (*n*=9), and Germany (*n*=5). Among the top 10 most-cited articles, both Sweden and the USA had three studies each. Moreover, the United States and Sweden stood at the forefront of global collaborations, each engaging with three distinct countries (Fig. 2c). Thirty-one institutions contributed to the research field. Karolinska Institutet in Sweden was the leading institute responsible for 24% of the spine publications among the top 100 most cited XR publications in the field of neurosurgery.

#### Study design and area of focus

Most spine publications were original research studies (89%), with only a minority being reviews (11%). A quarter of the publications addressed minimally invasive spine surgery techniques (24%). Most publications involved the use of AR (78%), followed by VR (19%), while MR was not explored. In contrast to VR which remained relatively stable (*p*=0.409), there was a significant increase in the number of publications on the use of AR (*p*=0.012) over time. The utilization of HMDs for the display of virtual elements was reported in ten studies, with Microsoft HoloLens being the most frequently used (40%).

Among the top cited spine only publications, XR was used on human subjects (35%), or in conjunction with physical 3D models (24%), cadavers (19%), virtual 3D models (11%), and screens (2.7%). The subject type was not reported in six studies (16%).

The applications of XR included neuronavigation (87%), surgical education (19%), assessing surgical skills (8.1%), and preoperative planning of surgical procedures (2.7%). The use of XR for neuronavigation experienced an increasing trend over the years (*p*=0.014), while other applications remained relatively stable (*p*≥0.05).

The specific procedures studied included pedicle screw placement (60%), vertebroplasty (11%), osteotomy (5.4%), discectomy (2.2%), tumor resection (2.2%), foraminotomy (2.2%), hemilaminectomy (2.2%), kyphoplasty (2.2%), and lumbar decompression (2.2%).

### Cranial only publications

Among the top 100 most cited XR publications within neurosurgery, 47 publications exclusively addressed cranial neurosurgery. There was no significant trend in publications over the years (*p*=0.746). However, the number of publications exhibited a temporary increase from 1998 to 2017 before tapering out during the next five years (Fig. 1).

Among the 47 publications, only 15 were open access (32%). The total citation count ranged from 24 to 191 citations, with a median count of 40.0 (IQR 29.0 – 59.5). The median number of citations per year was 5.2 (IQR 3.8 – 8.0). The most cited article was "Planning and simulation of neurosurgery in a virtual reality environment" by Kockro et al. [28], published in Neurosurgery in 2000 (Table 4). However, the article with the highest number of citations per year was "Impact of Virtual and Augmented Reality Based on Intraoperative Magnetic Resonance Imaging and Functional Neuronavigation in Glioma Surgery Involving Eloquent Areas" by Sun et al. [38], published in World Neurosurgery in 2016. On average, the article has been cited 17 times yearly. A significant increase in open access publishing per year was detected (*p*=0.025). No such trends were observed regarding the number of studies per year (*p*=0.746), and average citations/year (*p*=0.917).

**Table 4:** Top 10 studies addressing the use of extended reality in cranial neurosurgery

Rank	Year	Title	Citation count	Citations per year	First Author	Country
1	2000	Planning and simulation of neurosurgery in a virtual reality environment	191	6.96	Kockro	Singapore
2	2007	Virtual reality in neurosurgical education: Part-task ventriculostomy simulation with dynamic visual and haptic feedback	158	8.82	Lemole	USA
3	2012	NeuroTouch: A Physics-Based Virtual Simulator for Cranial Microneurosurgery Training	156	12.83	Delorme	Canada
4	2012	Neuronavigation in the surgical management of brain tumors: current and future trends	142	11.08	Orringer	USA
5	2008	Virtual reality system for planning minimally invasive neurosurgery	120	6.38	Stadie	Germany
6	2018	Clinical Feasibility of a Wearable Mixed-Reality Device in Neurosurgery	86	13.17	Incekara	Netherlands
7	2020	The Virtual Operative Assistant: An explainable artificial intelligence tool for simulation-based training in surgery and medicine	68	9.71	Mirchi	Canada
8	2013	The Development of a Virtual Simulator for Training Neurosurgeons to Perform and Perfect Endoscopic Endonasal Transsphenoidal Surgery	65	5.82	Rosseau	USA
9	2003	Computer-enhanced stereoscopic vision in a head-mounted operating binocular	64	8.8	Birkfellner	Switzerland
10	2013	Interactive presurgical simulation applying advanced 3D imaging and modeling techniques for skull base and deep tumors Clinical article	63	5	Oishi	Japan

#### Journal

A total of 24 journals contributed to the cranial only publications among the top 100 publications on XR within the field of neurosurgery. The top five journals in terms of contributions were Neurosurgery (*n*=9), followed by World Neurosurgery (*n*=6), International Journal of Computer Assisted Radiology and Surgery (*n*=4), Journal of Neurosurgery (*n*=3), and Operative Neurosurgery (*n*=2) (Table 3). Impact factors ranged from 0.98 to 13.83, with a median value of 3.42 (IQR 2.21 – 5.32).

#### Authorship

Forty-three different first authors and 38 last authors contributed to the top-cited cranial publications. The leading first authors were Kockro (*n*=3), Alotaibi (*n*=2), Azarnoush (*n*=2), Winkler-Schwartz (*n*=2), and Stadie (*n*=2). Notably, only two last authors were associated with two or more publications, namely Del Maestro (*n*=11) and Samii (*n*=2; Table 3). The majority of first authors (64%) and last authors (75%) held neurosurgical backgrounds.

The representation of female authors was limited, with only four first authors (8.5%) being females. There were no significant trends in terms of the proportion of female first authorship over the years (*p*=0.137). Only one last author was female. Furthermore, no significant increase in the number of female authors was identified in either first (*p*=0.917) or last (*p*=0.311) authorships. The total number of authors per publication ranged from 2 to 14, with an average of 7.06 authors per article (SD 2.9).

#### Global distribution

Thirteen countries participated in research on the application of XR in cranial surgery, with seven of them contributing two or more publications (Fig. 2b). Canada emerged as the leading country of origin (*n*=13), followed by China (*n*=7) and the USA (*n*=7). Furthermore, the USA played a central role in international collaboration. Intercontinental collaborations involving institutions in the Middle East for instance were particularly evident in cranial research (Fig. 2d). Thirty-eight institutions contributed to the research field. The most actively involved institution was McGill University in Canada, contributing 24% of the current literature.

#### Study design and area of focus

Most articles were original research studies (89%), covering a diverse range of neurosurgical subspecialities. Research topics included neurosurgical oncology (57%), skull base surgery (32%), vascular surgery (19%), and functional neurosurgery (4.2%). A subset of articles (6.4%) specifically investigated the application of XR for the placement of catheters for cerebrospinal fluid drainage.

Most publications involved the use of VR (49%), followed by AR (40%), and MR (4.3%). Moreover, studies on the use of both AR (*p*=0.025) and VR (*p*=0.041) have shown significant increases among cranial studies. However, a trend analysis of the past ten years illustrated that research regarding AR use (*p*=0.046) has increased while VR research was relatively steady (*p*=0.247). Eight studies utilized HMDs to visualize virtual elements, with Microsoft HoloLens being the most frequently used (*n*=2).

The applications of XR encompassed neuronavigation (64%), preoperative planning of surgical procedures (34%), surgical education (32%), and assessing surgical skills (23%). The use of XR for assessing surgical skills (*p*=0.007) and surgical education (*p*=0.008) experienced an increasing trend over the years, while other applications remained relatively stable (*p*≥0.05).

XR was used on virtual 3D models (36%), physical 3D models (30%), human subjects (19%), cadavers (4.3%), and screens (2.1%). The exact type of experimental subject was not reported in 10 studies (21%). Procedures explored in these studies included intracranial tumor resection (71%), endoscopic surgery (11%), aneurysm surgery (8.5%), AVM surgery (4.3%), craniotomy (4.3%), AVF resection (2.1%), DBS electrode implantation (2.1%), trigeminal rhizotomy (2.1%), and ventriculostomy (2.1%).

### Comparison between spinal and cranial publications

Several noteworthy distinctions between spinal and cranial publications were identified (Table 5). The number of spinal publications has experienced a significant increase, whereas cranial publications have seen a decline in the number of yearly publications over the past five years (Fig. 1). No difference regarding median citaions (*p*=0.528) and median average yearly citations (*p*=0.896) was significant. No differences regarding the proportion of female first and last authors were found (*p*=1). An equal proportion of original reseach articles was present in both groups (89%).

**Table 5:** Comparison between spine only and cranial only studies

Variable	Spine only (*N*=37)	Cranial only (*N*=47)	*p*-value*
Number of citations			0.528
Median [Q1, Q3]	38.0 [29.0, 58.0]	40.0 [29.0, 59.5]	
Citations per year			0.896
Median [Q1, Q3]	5.80 [2.77, 8.86]	5.20 [3.82, 8.00]	
Open access	18 (49%)	15 (32%)	0.182
Journal Impact Factor			0.655
Median [Q1, Q3]	3.24 [2.82, 3.62]	3.42 [2.21, 5.32]	
Female sex			1
First author	3 (8.1%)	4 (8.5%)	
Last author	0 (0%)	1 (2.1%)	
First author profession			NA
Neurosurgeon	19 (52%)	30 (64%)	
Engineer	6 (16%)	7 (15%)	
Orthopedic surgeon	5 (14%)	0 (0%)	
Last author profession			NA
Neurosurgeon	18 (49%)	35 (75%)	
Orthopedic surgeon	13 (35%)	0 (0%)	
Engineer	2 (5.4%)	8 (17%)	
Number of authors			0.887
Mean (SD)	7.08 (2.9)	7.06 (2.9)	
Article type			1
Article	33 (89%)	42 (89%)	
Review	4 (11%)	5 (11%)	
Models used			0.071
Human subjects	13 (35%)	9 (19%)	
Physical 3D models	9 (24%)	14 (30%)	
Cadavers	7 (18%)	2 (4.3%)	
Not mentioned	6 (16%)	10 (21%)	
Virtual 3D models	4 (11%)	17 (36%)	
Screen	1 (2.7%)	1 (2.1%)	
Type of XR			**0.007**
AR	29 (78%)	19 (40%)	
VR	7 (19%)	23 (49%)	
MR	0 (0%)	2 (4.3%)	
Main use of XR			**0.036**
Navigation	32 (87%)	30 (64%)	
Education and simulation	7 (19%)	15 (32%)	
Assessing surgical skills	3 (8.1%)	11 (23%)	
Surgical planning	1 (2.7%)	16 (34%)	
HMD	10 (27%)	8 (17%)	0.601

*Statistical comparison between cranial and spine only studies

Twenty-four countries and 31 institutions contributed to spinal publications, while 13 countries and 38 instiutions contributed to cranial publications. Both spinal and cranial studies predominantly took place in North America, but cranial research displayed more collaborations with Middle Eastern countries, whereas spinal publications predominantly involved collaboration between North America and Western Europe.

Spinal publications exhibited a distinct emphasis on AR applications, while cranial publications explored VR applications (*p*=0.007). Even though neuronavigation was the main use analyzed in both publication types, cranial studies showcased a heightened focus on education, surgical planning, and the assessment of surgical skills (*p*=0.036). Trend analysis illustrated an increase in spinal publications on neuronavigation (*p*=0.014), surgical education (p=0.008) and assessing surgical skills (*p*=0.007).

There were 16 publications that addressed both cranial and spinal surgery, the median citation count was 50.5 [37.8, 72.3], and the median of the average yearly citations was 7.36 [IQR 5.41 – 12.2]. Most articles were original contributions (63%), with 31% available as open access. In comparison to publications exclusively focused on cranial or spinal surgeries, a higher proportion (37%) were review articles. Only one female author held the position of first author, and none were last author. Publications encompassed analyses of AR (50%), VR (31%), and MR (13%). The diverse range of uses explored included surgical education (57%), neuronavigation (38%), and patient satisfaction (6.3%).

## Discussion

The findings of this study underscore a notable increase in publications related to XR in neurosurgery, indicative of the growing interest in the potential advantages offered by this technology in the improvement of neurosurgical procedures and education and training of surgeons [7, 8, 17, 31, 32]. Through this bibliometric analysis, we analyzed the top 100 most impactful and widely cited publications on different applications of XR within the field of neurosurgery.

The increase in yearly citations for spinal publications was matched by a substantial rise in open-access publishing, which may have had a positive impact on the number of citations [13]. Despite increased open-access in cranial publications, there was no corresponding increase in citation metrics. Moreover, there was a decrease in the yearly rate of cranial publications during the last 5 years studied.

There were no differences in impact factors between spinal and cranial publications. Most journals had impact factors between 2.2 and 5.2, which aligns with the range observed in neurosurgical journals, spanning from 2.1 to 5.1 [26, 37].

The greater number of authors, institutions, and countries contributing to the top cited publications on XR in cranial neurosurgery suggests a more diversified field. In spine surgery, publications originated from fewer author groups, institutions, and countries. While applications in spine surgery were mostly focused around neuronavigation, applications in cranial surgery included surgical skill assessment, surgical planning, education, and simulation, as well as navigation.

Female first and last authorship in neurosurgery is estimated at 12% of articles [4]. A comparable but lower representation of female authorship was found in this study [35]. The low representation also contrasts with a prior bibliometric examination focused on the application of artificial intelligence in neurosurgery which did not observe differences of such magnitude [18]. Beyond simply reflecting the underlying differences in the scientific field, the poor representation of female authors may also impact the focus and direction of future developments [34, 35]. However, over the past two decades, there has been a significant increase in the number of female medical students [30], a trend that is expected to be reflected in future neurosurgical publications. Early enrollment of medical students in neuronavigational research could help minimize current gender disparities in the future.

The worldwide distribution of contributing countries, along with variations in collaborations, may be correlated with regional interests and levels of technological development. The noticeable absence of contributions from developing countries is most likely attributable to limited resources [27]. Northern America emerges as a significant contributor to the existing body of literature, spanning both spinal and cranial procedures. Western Europe, on the other hand, exhibits a focus on spinal implementation. Additionally, the landscape of collaborations reveals a greater participation of East-Asian countries in spinal procedures compared to their West-Asian counterparts.

While developing countries currently lag in adopting augmented reality technology, XR holds promise for advancing neurosurgical practice within these regions. Several centers have developed affordable and portable XR systems that could be employed for neuronavigation in low resource settings [2, 9, 15, 23]. Additionally, tele-mentoring is a notable feature of XR. In numerous cities across the African continent, there is a shortage of neurosurgeons [10]. Through virtual reality guidance, neurosurgeons from neighboring cities or countries can remotely provide live feedback during life-saving neurosurgical procedures [16].

Our analysis reveals a significant interest in the application of AR for neuronavigation in spinal surgery, while VR dominates in the realm of surgical training for cranial procedures. Even though AR research is increasing in cranial procedures, a higher proportion of VR publications was identified in cranial publications.

The focus on VR research in cranial neurosurgery reflected studies on surgical planning, training, and assessment of surgical skills. In this context neuro-oncological and skull base procedures emerged as the most frequently studied cranial procedures. The use of VR models for training and assessing surgical skills provides simulation cases which can be used repeatedly. AR research was concentrated on developing neuronavigation solutions. However, in cranial neurosurgery procedures, heads-up displays, commonly used for AR overlay, must consider the surgical microscope [40]. Thus, only a limited number of cranial procedures, such as external ventricular drain placements, are performed without a surgical microscope and easily converted to AR-guided procedures using HMD. In contrast to the malleable brain anatomy, the bony anatomy of the spine facilitates the implementation of AR solutions [14, 39]. Among these, AR studies on the accurate placement of pedicle screws represent a great part of the studies. The clinical challenges and the maturity of the technological solutions differ between cranial and spinal neurosurgery and these differences are reflected in scientific literature. Cranial navigation has been part of routine practice for decades while the corresponding implementation in spine surgery is still lacking [6].

The staggering number of cranial publications can be attributed to the well-established neuronavigational workflow adopted across various neurosurgical clinics. The inception of research in cranial neuronavigation predates that of spinal surgery [20]. Our findings indicate a predominant focus on cranial applications in the earlier stages of utilizing XR in neurosurgery. This early emphasis on developing navigational solutions for cranial procedures has contributed to the current established workflow. The presence of a functional neuronavigational solution may have hindered a sustained interest in further research regarding XR solutions. This phenomenon could explain why the most cited cranial publications show a lag in publication years compared to spinal studies. The top two articles in spinal publications were published in 2013 and 2019, whereas the leading cranial publications date back to 2000 and 2007. Moreover, it can be argued that outdated research and technology may still be cited in cranial studies, which may lack relevance for contemporary medical practices. This raises concerns about the applicability of older findings and methodologies in the current landscape of neurosurgery.

It is crucial to emphasize that this article does not delve into recent original research that may currently be overlooked. Arguably, the more innovative the application of technology, the less likely it is to be included in this list, as novel techniques often require time for widespread acceptance. An illustrative case in point is the utilization of VR for cranial anatomic research [43]. VR serves as the sole means by which we can manipulate the eye for transorbital exposure research. It provides a unique avenue for comparing how tumors themselves impact surgical freedom across various approaches and enables a comprehensive evaluation of surgical exposure by comparing different approaches with overlapping bone removal [33]. Despite the evident value of these applications, it appears that the filed is not receiving adequate attention, let alone citations. This observation may suggest that readers are still in the process of familiarizing themselves with these advancements.

### Limitations

It is important to note that the conclusions of this analysis only reflect the top-cited articles without considering the total number of submissions and publications relevant to this field. Also, the ranking presented in this work solely relies on the Web of Science citation metric system, which may differ from others such as Google Scholar, Scopus, or PubMed. However, this issue has previously been described, and a method to report citation counts more accurately, for all the existing databases, has yet to be described [18]. Moreover, determining the scientific value and impact of papers based on citation metrics has limited validity.

## Conclusion

In conclusion, the field of neurosurgery is witnessing a substantial increase in research focused on the application of XR, particularly in spinal neurosurgery, where the use of AR for navigation is gaining attention. The USA and Sweden were behind most of the highest cited studies on XR in spinal neurosurgery. Meanwhile, in cranial neurosurgery XR-research has increasingly addressed surgical planning and training using VR applications. However, in recent years a shift towards AR was noticed. Most of the top cited studies on XR in cranial neurosurgery originated from North America.

## Supplementary information

Below is the link to the electronic supplementary material.

Supplementary file1 (DOCX 40 KB)

## Data Availability

The data utilized to conduct the analyses can be provided by the corresponding author upon reasonable request.

## Abbreviations

AR: augmented reality
VR: virtual reality
MR: mixed reality
XR: extended reality
HMD: head-mounted display

## Code availability

A qmd file containing the R code written to conduct the analyses can be provided by the corresponding author upon reasonable request.
