# Supplementary material for: Extended reality in cranial and spinal neurosurgery – a bibliometric analysis
Source: Acta Neurochir (Wien). 2024 Apr 25;166(1):194. doi: 10.1007/s00701-024-06072-4 (PMC11045579; doi:10.1007/s00701-024-06072-4)
Supplement: Supplementary file 1 — Supplementary file1 (DOCX 40 KB) [file 701_2024_6072_MOESM1_ESM.docx]

**Supplementary Table 1. Results of bibliometric search.** The top 100 most-cited articles are listed in order of most citations.

| **Rank** | **Spinal/**  **Cranial** | **Article** | **First author** | **Total Citations** | **Average yearly citation** |
| --- | --- | --- | --- | --- | --- |
| 1 | Cranial | Planning and simulation of neurosurgery in a virtual reality environment | Kockro, RA | 191 | 6.96 |
| 2 | Both | Augmented reality in neurosurgery: a systematic review | Meola, Antonio | 162 | 22.86 |
| 3 | Cranial | Virtual reality in neurosurgical education: Part-task ventriculostomy simulation with dynamic visual and haptic feedback | Lemole, G. Michael, Jr. | 158 | 8.82 |
| 4 | Cranial | NeuroTouch: A Physics-Based Virtual Simulator for Cranial Microneurosurgery Training | Delorme, Sebastien | 156 | 12.83 |
| 5 | Both | Utilizing virtual and augmented reality for educational and clinical enhancements in neurosurgery | Pelargos, Panayiotis E. | 151 | 21.14 |
| 6 | Cranial | Neuronavigation in the surgical management of brain tumors: current and future trends | Orringer, Daniel A. | 142 | 11.08 |
| 7 | Cranial | Virtual reality system for planning minimally invasive neurosurgery | Stadie, Axel Thomas | 120 | 6.38 |
| 8 | Spinal | A novel 3D guidance system using augmented reality for percutaneous vertebroplasty | Abe, Yuichiro | 109 | 7.45 |
| 9 | Spinal | Pedicle Screw Placement Using Augmented Reality Surgical Navigation With Intraoperative 3D Imaging A First In-Human Prospective Cohort Study | Elmi-Terander, Adrian | 106 | 21 |
| 10 | Spinal | Augmented reality navigation with intraoperative 3D imaging vs fluoroscopy-assisted free-hand surgery for spine fixation surgery: a matched-control study comparing accuracy | Elmi-Terander, Adrian | 103 | 10 |
| 11 | Spinal | Head-mounted display augmented reality to guide pedicle screw placement utilizing computed tomography | Gibby, Jacob T. | 95 | 18.8 |
| 12 | Both | Virtual reality-based simulators for spine surgery: a systematic review | Pfandler, Michael | 88 | 12 |
| 13 | Cranial | Clinical Feasibility of a Wearable Mixed-Reality Device in Neurosurgery | Incekara, Fatih | 86 | 13.17 |
| 14 | Both | Role of Cranial and Spinal Virtual and Augmented Reality Simulation Using Immersive Touch Modules in Neurosurgical Training | Alaraj, Ali | 73 | 6.27 |
| 15 | Both | Effect of an Immersive Preoperative Virtual Reality Experience on Patient Reported Outcomes A Randomized Controlled Trial | Bekelis, Kimon | 72 | 17 |
| 16 | Spinal | Feasibility and Accuracy of Thoracolumbar Minimally Invasive Pedicle Screw Placement With Augmented Reality Navigation Technology | Elmi-Terander, Adrian | 71 | 11.17 |
| 17 | Spinal | Learning Retention of Thoracic Pedicle Screw Placement Using a High-Resolution Augmented Reality Simulator With Haptic Feedback | Luciano, Cristian J. | 70 | 5.31 |
| 18 | Cranial | The Virtual Operative Assistant: An explainable artificial intelligence tool for simulation-based training in surgery and medicine | Mirchi, Nykan | 68 | 9.71 |
| 19 | Spinal | Augmented reality surgical navigation with ultrasound-assisted registration for pedicle screw placement: a pilot study | Ma, Longfei | 67 | 8.86 |
| 20 | Cranial | The Development of a Virtual Simulator for Training Neurosurgeons to Perform and Perfect Endoscopic Endonasal Transsphenoidal Surgery | Rosseau, Gail | 65 | 5.82 |
| 21 | Cranial | Computer-enhanced stereoscopic vision in a head-mounted operating binocular | Birkfellner, W | 64 | 8.8 |
| 22 | Cranial | Interactive presurgical simulation applying advanced 3D imaging and modeling techniques for skull base and deep tumors Clinical article | Oishi, Makoto | 63 | 5 |
| 23 | Cranial | DEX-RAY: AUGMENTED REALITY NEUROSURGICAL NAVIGATION WITH A HANDHELD VIDEO PROBE | Kockro, Ralf A. | 60 | 3.93 |
| 24 | Both | Virtual and stereoscopic anatomy: when virtual reality meets medical education | Vieira de Faria, Jose Weber | 60 | 7.38 |
| 25 | Cranial | Machine Learning Identification of Surgical and Operative Factors Associated With Surgical Expertise in Virtual Reality Simulation | Winkler-Schwartz, Alexander | 60 | 11.8 |
| 26 | Cranial | Neurosurgical virtual reality simulation metrics to assess psychomotor skills during brain tumor resection | Azarnoush, Hamed | 59 | 6.44 |
| 27 | Spinal | Clinical Accuracy, Technical Precision, and Workflow of the First in Human Use of an Augmented-Reality Head-Mounted Display Stereotactic Navigation System for Spine Surgery | Molina, Camilo A. | 59 | 11.4 |
| 28 | Both | Virtual reality neurosurgery: A simulator blueprint | Spicer, MA | 59 | 12.75 |
| 29 | Spinal | Virtual reality spine surgery simulation: an empirical study of its usefulness | Gasco, Jaime | 58 | 5.4 |
| 30 | Cranial | Assessing performance in brain tumor resection using a novel virtual reality simulator | Gelinas-Phaneuf, Nicholas | 58 | 2.07 |
| 31 | Spinal | Real-time advanced spinal surgery via visible patient model and augmented reality system | Wu, Jing-Ren | 58 | 2.43 |
| 32 | Both | IBIS: an OR ready open-source platform for image-guided neurosurgery | Drouin, Simon | 57 | 8.14 |
| 33 | Cranial | Image-Guided Neurosurgery With 3-Dimensional Multimodal Imaging Data on a Stereoscopic Monitor | Kockro, Ralf A. | 55 | 7 |
| 34 | Cranial | Augmented reality in neurovascular surgery: feasibility and first uses in the operating room | Kersten-Oertel, Marta | 53 | 10 |
| 35 | Spinal | Augmented reality navigation in spine surgery: a systematic review | Burström, Gustav | 52 | 5.56 |
| 36 | Cranial | A new head-mounted display-based augmented reality system in neurosurgical oncology: a study on phantom | Cutolo, Fabrizio | 50 | 4.9 |
| 37 | Cranial | Assessing Bimanual Performance in Brain Tumor Resection With NeuroTouch, a Virtual Reality Simulator | Alotaibi, Fahad E. | 47 | 5.11 |
| 38 | Cranial | Smart Glasses for Neurosurgical Navigation by Augmented Reality | Maruyama, Keisuke | 47 | 7.83 |
| 39 | Cranial | Virtual reality augmentation in skull base surgery | Rosahl, SK | 47 | 7.6 |
| 40 | Spinal | Augmented reality and artificial intelligence-based navigation during percutaneous vertebroplasty: a pilot randomised clinical trial | Auloge, Pierre | 46 | 11.25 |
| 41 | Spinal | Percutaneous Spinal Fixation Simulation With Virtual Reality and Haptics | Luciano, Cristian J. | 45 | 10.75 |
| 42 | Spinal | Augmented reality navigation for spinal pedicle screw instrumentation using intraoperative 3D imaging | Mueller, Fabio | 45 | 11.25 |
| 43 | Cranial | Virtual reality presurgical planning for cerebral gliomas adjacent to motor pathways in an integrated 3-D stereoscopic visualization of structural MRI and DTI tractography | Qiu, Tian-ming | 45 | 4.67 |
| 44 | Both | Implementation of augmented reality support in spine surgery | Carl, Barbara | 44 | 7.33 |
| 45 | Spinal | Image guided percutaneous spine procedures using an optical see-through head mounted display: proof of concept and rationale | Deib, Gerard | 44 | 2.1 |
| 46 | Spinal | Surgical Navigation Technology Based on Augmented Reality and Integrated 3D Intraoperative Imaging: A Spine Cadaveric Feasibility and Accuracy Study | Elmi-Terander, Adrian | 43 | 3.91 |
| 47 | Cranial | Proficiency Performance Benchmarks for Removal of Simulated Brain Tumors Using a Virtual Reality Simulator NeuroTouch | AlZhrani, Gmaan | 42 | 3 |
| 48 | Spinal | Artificial Intelligence Distinguishes Surgical Training Levels in a Virtual Reality Spinal Task | Bissonnette, Vincent | 42 | 8.2 |
| 49 | Both | Impact of a self-developed planning and self-constructed navigation system on skull base surgery: 10 years experience | Caversaccio, Marco | 41 | 2.85 |
| 50 | Both | Intraoperative clinical application of augmented reality in neurosurgery: A systematic review | Contreras Lopez, William Omar | 41 | 5.13 |
| 51 | Cranial | Impact of Virtual and Augmented Reality Based on Intraoperative Magnetic Resonance Imaging and Functional Neuronavigation in Glioma Surgery Involving Eloquent Areas | Sun, Guo-chen | 41 | 16.5 |
| 52 | Cranial | Augmented reality-assisted skull base surgery | Cabrilo, I. | 40 | 3.55 |
| 53 | Spinal | A Novel Augmented-Reality-Based Surgical Navigation System for Spine Surgery in a Hybrid Operating Room: Design, Workflow, and Clinical Applications | Edström, Erik | 40 | 5.71 |
| 54 | Cranial | Brain Tumor Surgery With 3-Dimensional Surface Navigation | Mert, Ayguel | 40 | 6.6 |
| 55 | Spinal | Machine learning for automated 3-dimensional segmentation of the spine and suggested placement of pedicle screws based on intraoperative cone-beam computer tomography | Burström, Gustav | 39 | 9.75 |
| 56 | Both | Enhancing Reality: A Systematic Review of Augmented Reality in Neuronavigation and Education | Cho, James | 38 | 9 |
| 57 | Spinal | Augmented reality-assisted pedicle screw insertion: a cadaveric proof-of-concept study | Molina, Camilo A. | 38 | 2.24 |
| 58 | Cranial | Registration and fusion quantification of augmented reality based nasal endoscopic surgery | Chu, Yakui | 37 | 5.14 |
| 59 | Both | Development of a Mixed Reality Platform for Lateral Skull Base Anatomy | McJunkin, Jonathan L. | 37 | 5.5 |
| 60 | Cranial | Intraoperative stereoscopic QuickTime Virtual Reality | Balogh, A | 36 | 10.67 |
| 61 | Spinal | Augmented Reality Surgical Navigation in Spine Surgery to Minimize Staff Radiation Exposure | Edström, Erik | 36 | 2.77 |
| 62 | Cranial | Stereoscopic navigation-controlled display of preoperative MRI and intraoperative 3D ultrasound in planning and guidance of neurosurgery: New technology for minimally invasive image-guided surgery approaches | Hernes, TAN | 36 | 5.5 |
| 63 | Cranial | Virtual reality and augmented reality in the management of intracranial tumors: A review | Lee, Chester | 36 | 2 |
| 64 | Both | Virtual Reality in Neurosurgery: Beyond Neurosurgical Planning | Mishra, Rakesh | 36 | 2.77 |
| 65 | Cranial | Bimanual Psychomotor Performance in Neurosurgical Resident Applicants Assessed Using NeuroTouch, a Virtual Reality Simulator | Winkler-Schwartz, Alexander | 36 | 4.38 |
| 66 | Spinal | Navigation and Image Injection for Control of Bone Removal and Osteotomy Planes in Spine Surgery | Kosterhon, Michael | 35 | 7.75 |
| 67 | Spinal | Robotic Spine Surgery and Augmented Reality Systems: A State of the Art | Vadala, Gianluca | 34 | 10.67 |
| 68 | Cranial | Presurgical and Intraoperative Augmented Reality in Neuro-Oncologic Surgery: Clinical Experiences and Limitations | Mikhail, Mirriam | 33 | 1.57 |
| 69 | Cranial | Augmented Reality-Assisted Craniotomy for Parasagittal and Convexity En Plaque Meningiomas and Custom-Made Cranio-Plasty: A Preliminary Laboratory Report | Montemurro, Nicola | 32 | 8 |
| 70 | Cranial | Impact of acute stress on psychomotor bimanual performance during a simulated tumor resection task | Bajunaid, Khalid | 31 | 4.43 |
| 71 | Both | Mixed-Reality Simulation for Neurosurgical Procedures | Bova, Frank J. | 31 | 1.75 |
| 72 | Spinal | Microscope-Rased Augmented Reality in Degenerative Spine Surgery: Initial Experience | Carl, Barbara | 31 | 6 |
| 73 | Both | Virtual Reality in Neurosurgery: Can You See It?-A Review of the Current Applications and Future Potential | Fiani, Brian | 31 | 6.2 |
| 74 | Spinal | A cadaveric precision and accuracy analysis of augmented reality-mediated percutaneous pedicle implant insertion | Molina, Camilo A. | 31 | 6 |
| 75 | Cranial | Usefulness of a Virtual Reality Percutaneous Trigeminal Rhizotomy Simulator in Neurosurgical Training | Shakur, Sophia F. | 31 | 5 |
| 76 | Spinal | Augmented reality-based navigation increases precision of pedicle screw insertion | Dennler, Cyrill | 30 | 5.8 |
| 77 | Cranial | Virtual Reality-Based Simulators for Cranial Tumor Surgery: A Systematic Review | Mazur, Travis | 30 | 2.14 |
| 78 | Spinal | Workflow Caveats in Augmented Reality-Assisted Pedicle Instrumentation: Cadaver Lab | Urakov, Timur M. | 30 | 3.33 |
| 79 | Spinal | Augmented reality in intradural spinal tumor surgery | Carl, Barbara | 29 | 1.71 |
| 80 | Spinal | Evolving Navigation, Robotics, and Augmented Reality in Minimally Invasive Spine Surgery | Hussain, Ibrahim | 29 | 5.8 |
| 81 | Spinal | An augmented reality system for image-guided surgery | Marmulla, R | 29 | 1.47 |
| 82 | Spinal | The utility of virtual reality and augmented reality in spine surgery | Yoo, Joon S. | 29 | 2.64 |
| 83 | Cranial | A Novel Augmented Reality Navigation System for Endoscopic Sinus and Skull Base Surgery: A Feasibility Study | Li, Liang | 28 | 8.33 |
| 84 | Cranial | Neurosurgical craniotomy localization using a virtual reality planning system versus intraoperative image-guided navigation | Stadie, Axel T. | 28 | 6.5 |
| 85 | Cranial | Image-guided lateral suboccipital approach: Part 2 - Impact on complication rates and operation times | Gharabaghi, Alireza | 27 | 4.17 |
| 86 | Spinal | Artificial Neural Networks to Assess Virtual Reality Anterior Cervical Discectomy Performance | Mirchi, Nykan | 27 | 1 |
| 87 | Cranial | Clinical evaluation and follow-up outcome of presurgical plan by Dextroscope: a prospective controlled study in patients with skull base tumors | Yang, De L. | 27 | 5.2 |
| 88 | Cranial | The force pyramid: a spatial analysis of force application during virtual reality brain tumor resection | Azarnoush, Hamed | 26 | 1.73 |
| 89 | Cranial | Endoscopic navigation system with extended field of view using augmented reality technology | Bong, Jae Hwan | 26 | 3.13 |
| 90 | Cranial | Neurosurgical Virtual Reality Simulation for Brain Tumor Using High-definition Computer Graphics: A Review of the Literature | Kin, Taichi | 26 | 8 |
| 91 | Cranial | Virtual reality technology for teaching neurosurgery of skull base tumor | Shao, Xuefei | 26 | 3.71 |
| 92 | Spinal | The Efficacy of Immersive Virtual Reality Surgical Simulator Training for Pedicle Screw Placement: A Randomized Double-Blind Controlled Trial | Xin, Baoquan | 26 | 2 |
| 93 | Cranial | Neurosurgical Assessment of Metrics Including Judgment and Dexterity Using the Virtual Reality Simulator NeuroTouch (NAJD Metrics) | Alotaibi, Fahad E. | 25 | 4.8 |
| 94 | Cranial | Next-Generation Surgical Navigation Systems in Sinus and Skull Base Surgery | Citardi, Martin J. | 25 | 1.56 |
| 95 | Spinal | Accuracy of augmented reality surgical navigation for minimally invasive pedicle screw insertion in the thoracic and lumbar spine with a new tracking device | Peh, Simon | 25 | 6 |
| 96 | Cranial | Virtual Reality Angiogram vs 3-Dimensional Printed Angiogram as an Educational tool-A Comparative Study | Bairamian, David | 24 | 2.67 |
| 97 | Cranial | Virtual endoscopy (VE) of the basal cisterns: Its value in planning the neurosurgical approach | Boor, S | 24 | 0.92 |
| 98 | Spinal | The Role of Mixed Reality Simulation for Surgical Training in Spine Phase 1 Validation | Coelho, Giselle | 24 | 2.4 |
| 99 | Spinal | Augmented and virtual reality in spine surgery, current applications and future potentials | Ghaednia, Hamid | 24 | 3.43 |
| 100 | Spinal | Augmented and Virtual Reality Instrument Tracking for Minimally Invasive Spine Surgery | Burström, Gustav | 23 | 7.67 |
